# Clinical and laboratorial factors and their relation to asthma severity

**DOI:** 10.1186/1939-4551-8-S1-A45

**Published:** 2015-04-08

**Authors:** José Elabras Filho, Solange Oliveira Rodrigues Valle, Augusto Tiaqui Abe, Rosângela Prendim Tórtora, Ana Paula Ferracciú Coutinho Millet, Juliana Salvini Barbosa Martins Da Fonseca, Omar Lupi

**Affiliations:** 1 Hospital Universitário Clementino Fraga Filho Hucff-Ufrj, Brazil; 2 Hospital São Zacharias, Brazil

## Background

Many factors are related to asthma severity. We have investigated clinical-epidemiological and laboratorial findings in an asthmatic population of an University Hospital, and evaluated their impact for asthma severity.

## Patients and methods

We have studied 142 patients with asthma, diagnosed by clinical spirometric findings, attended at the Clinical Immunology outpatient service of an University Hospital. They were divided into two groups according to their asthma severity: group 1 (n=72), mild intermittent or mild persistent asthma, and group 2 (n=70) with moderate or severe asthma. They were submitted to medical history, physical examination and laboratorial routine tests, x-rays, spirometry and aeroallergens skin prick tests, and their findings were compared between both groups.

## Results

Patients mean age was 49.5 years-old, most of them were female and caucasian. 26.1% of them were former smokers. The majority also had rhinitis, positive aeroallergens skin prick tests, and atopy family history. The comparison of numerical variables between groups 1 and 2 was statistically significant for age (higher in group 2), and for absolute and % predicted peak expiratory flow (lower in group 2), and pre and post bronchodilator FEV 1 % predicted (lower in group 2). Comparisons of categorical variables between the two groups showed a significant difference in the prevalence of rhinitis (p<0.001), and a statistical trend for increase of eosinophil percentage, more common in group 1. We also observed in group 1 a statistical trend with the positivity of aeroallergens prick tests, and owning a pet. A statistical trend for hypertension, gastroesophageal reflux and hyper or hypothyroidism was observed in group 2.

## Conclusions

Contributed to asthma major worsening: age, worse lung function parameters (FEV1 and peak flow), gastroesophageal reflux, and hyper or hypothyroidism. Contributed to asthma minor worsening: rhinitis, hypereosinophilia, positive aeroallergens skin prick tests, and owning a pet.

